# Dissecting the role of His domain protein tyrosine phosphatase/PTPN23 and ESCRTs in sorting activated epidermal growth factor receptor to the multivesicular body

**DOI:** 10.1042/BST20170443

**Published:** 2018-09-06

**Authors:** Lydia Tabernero, Philip Woodman

**Affiliations:** School of Biological Sciences, Faculty of Biology Medicine and Health, University of Manchester, Manchester Academic Health Science Centre, Manchester, U.K.

**Keywords:** endosomal sorting, epidermal growth factor receptor, ESCRT, HD-PTP, MVB

## Abstract

Sorting of activated epidermal growth factor receptor (EGFR) into intraluminal vesicles (ILVs) within the multivesicular body (MVB) is an essential step during the down-regulation of the receptor. The machinery that drives EGFR sorting attaches to the cytoplasmic face of the endosome and generates vesicles that bud into the endosome lumen, but somehow escapes encapsulation itself. This machinery is termed the ESCRT (endosomal sorting complexes required for transport) pathway, a series of multi-protein complexes and accessory factors first identified in yeast. Here, we review the yeast ESCRT pathway and describe the corresponding components in mammalian cells that sort EGFR. One of these is His domain protein tyrosine phosphatase (HD-PTP/PTPN23), and we review the interactions involving HD-PTP and ESCRTs. Finally, we describe a working model for how this ESCRT pathway might overcome the intrinsic topographical problem of EGFR sorting to the MVB lumen.

## Introduction

**Figure 1.**
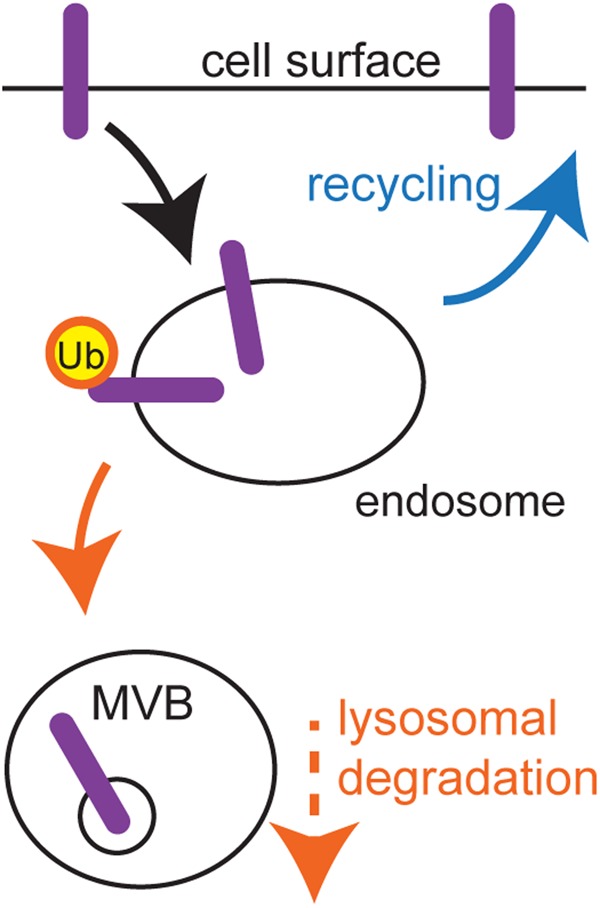
MVB sorting. A crucial decision is made in the endosome. Internalised plasma membrane proteins either recycle or are ubiquitinated and sorted to the MVB.

Plasma membrane proteins govern how cells sense their environment, and therefore, the surface levels of many of these proteins are subject to strict control. A key device for exerting such control is the endocytic pathway, and of particular importance is determining the fate of internalised membrane proteins once they enter the early endosome. Some proteins return to the surface and hence retain their function. Others are ubiquitinated and then sorted into vesicles that bud into the endosome lumen (intraluminal vesicles; ILVs) to form the multivesicular body (MVB), an intermediate compartment *en route* to the degradative milieu of the lysosome (Figure 1).

MVB sorting affects a host of cellular activities, but its importance is exemplified by how it controls epidermal growth factor-mediated signalling. Ligand-activated epidermal growth factor receptor (EGFR/ErbB1) is ubiquitinated by Cbl ubiquitin ligase [1] and then sorted to the MVB and thus down-regulated to turn off the signalling response. Defective MVB sorting of EGFR and other growth factor receptors is linked to tumour formation and common diseases [2–6]. Here, we review the mechanisms underlying MVB sorting, notably of EGFR.

## MVB sorting: endosomal sorting complexes required for transports

**Figure 2.**
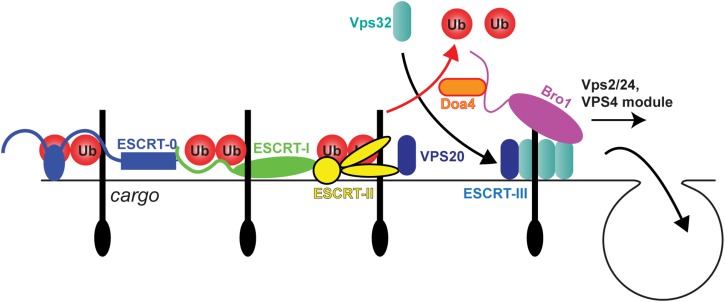
The ESCRT pathway drives MVB formation in yeast. ESCRT complexes act in succession to facilitate the MVB sorting of ubiquitinated membrane proteins.

The molecular machinery that drives MVB sorting is termed the endosomal sorting complexes required for transport (ESCRT) pathway, a series of multi-protein complexes first identified in the yeast *Saccharomyces cerevisiae* (Table 1 and Figure 2). Deletion of ESCRT components in yeast causes gross changes to endosome morphology [7,8] and prevents ubiquitinated cargo from being sorted to the lumen of the vacuole [9].

**Table 1:** Conservation of ESCRTs

	Yeast protein/alternative name	Human protein/alternative name
ESCRT-0	Hse1	STAM1,2
	Vps27	Hrs
ESCRT-I	Vps23/Stp22	TSG101/VPS23
	Vps28	VPS28
	Vps37	VPS37A,B,C,D
	Mvb12	MVB12A,B; UBAP1
ESCRT-II	Vps22/Snf8	EAP30
	Vps25	EAP20
	Vps36	EAP45
ESCRT-III	Vps2/Did4	CHMP2A,B
	Vps20	CHMP6
	Vps24	CHMP3
	Vps32/Snf7	CHMP4A,B,C
VPS4 module	Vps60	CHMP5
	Vps46/Did2	CHMP1A,B
	Vta1	VTA1
	Vps4	VPS4A,B
Accessory proteins	Vps31/Bro1	Alix; HD-PTP
	Doa4	UBPY/USP8

The yeast ESCRTs act sequentially (see Figure 2) [10–12]. The pathway begins with ESCRT-0, a dimer of Vps27 (vacuolar protein sorting 27) and Hse1 [Hrs-binding protein, STAM (signal transducing adaptor molecule) and EAST 1 (EGFR-associated protein with SH3 and TAM domain 1)] [13]. The multiple ubiquitin-binding domains (UBDs) within ESCRT-0 collectively generate a high avidity ubiquitin-binding module to sequester MVB cargo within a sub-domain of the endosome-limiting membrane [14]. Cargo is thought to then transfer from ESCRT-0 to ESCRT-I [15] (composed of Vps23, Vps28, Vps37 and MVB12 [MVB protein of 12 kDa] subunits) [16–18] and then on to ESCRT-II (comprising Vps36, Vps22 and two copies of Vps25) [16–19] (see Figure 2). ESCRT-I and -II both bind ubiquitin [15,20] and form a ‘super-complex’ on the endosome surface [21,22]. To complete this potentially linear ESCRT assembly pathway [23], ESCRT-II recruits ESCRT-III via the ‘initiator’ ESCRT-III subunit, Vps20 [24] (Vps20 can also be recruited by the Vps28 subunit of ESCRT-I [25]). Vps20 then seeds the formation of a membrane-sculpting polymer of another ESCRT-III subunit, Vps32 (also termed Snf7) [26–29]. Other ESCRT-III subunits (Vps2 and Vps24) then cap the polymer [28,29].

Aside from ESCRTs, other components are important for MVB formation. These include the accessory protein Vps31/Bro1 (Bck1-like resistance to osmotic shock 1) [30], which binds Vps32/Snf7 [31]; the deubiquitinase Doa4, which hydrolyses ubiquitin from cargo prior to completion of the ILV [32,33], and the VPS4 module [34]. This ATPase remodels, and ultimately disassembles, the ESCRT-III polymer to complete membrane scission and release monomeric ESCRT-III subunits back into the cytosol (see Figure 2).

## ESCRTs and EGFR sorting to the MVB

These ESCRT pathway constituents are broadly conserved. However, multiple variants of several components exist in mammalian cells (Table 1). For example, for mammalian ESCRT-I, there are single genes for the VPS23 [also called TSG101 (tumour susceptibility gene 101)] and VPS28 subunits, but four variants of VPS37 (VPS37A–D) and three variants of MVB12 (MVB12A/B and UBAP1) [17]. Mammalian cells have several Bro1 variants, notably Alix (ALG-2 [apoptosis-linked gene 2] interacting protein X) and HD-PTP (His domain protein tyrosine phosphatase) [17]. Aside from MVB sorting, ESCRTs support other ‘reverse-topology’ membrane scission events in the cell, including viral budding, cytokinesis, and resealing of the plasma membrane and nuclear envelope [16,17]. Therefore, a major challenge has been to establish which ESCRTs act at the endosome during EGFR sorting. While a consensus has not fully emerged, EGFR appears to follow at least two parallel ESCRT pathways, each co-ordinated by a specific Bro1 protein and perhaps subject to distinct EGFR-dependent signalling networks.

Both Alix and HD-PTP share with yeast Bro1 an N-terminal Bro1 domain that harbours a conserved binding pocket for the C-terminus of CHMP4B (charged membrane protein/chromatin modifying protein 4B) [31] (the most abundant mammalian variant of Vps32/Snf7; Table 1), a coiled-coil domain (which in Alix adopts a V-shaped structure and is termed the V domain [35,36]) and a proline-rich region (PRR). In HD-PTP, the PRR is particularly extensive and is followed by a PTP domain and a PEST region [37]. The PTP domain has no readily observable catalytic activity towards model PTPase substrates [38], but displays PTPase activity towards FYN kinase [39].

Alix supports multiple ESCRT-mediated processes [40]. While some studies have found siRNA-mediated depletion of Alix without effect on ligand-dependent EGF/EGFR degradation [41,42], others have observed modest [43] or more striking [44] reduction. Interestingly, Alix depletion significantly inhibited the MVB sorting of EGFR activated by UV irradiation, but not EGFR activated by EGF [45]. It is presently unclear which ESCRTs work alongside Alix during MVB formation. However, VPS37B and VPS37C, but not VPS37A, form complexes with Alix via Alg-2, an endosomal Ca^++^-dependent regulator of Alix [46]. VPS37B and C each form ESCRT-I complexes with MVB12A/B, both of which have been implicated in EGFR down-regulation [47]. Hence, a subset of ESCRT-I complexes might work alongside Alg-2 and Alix to promote a Ca^++^-regulated EGFR sorting pathway.

**Figure 3.**
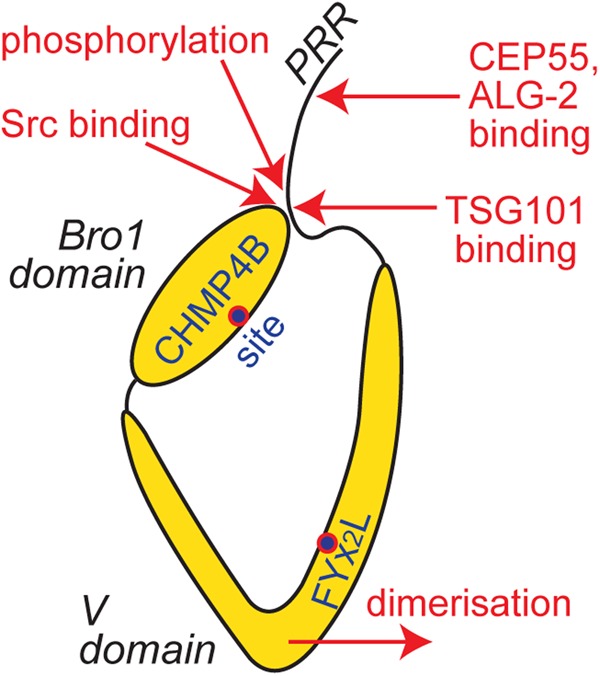
Alix is controlled by auto-inhibition and conformational change. Key binding sites within Alix Bro1 and V domains are made inaccessible by an auto-inhibitory interaction between the Bro1 domain and PRR. Several events, highlighted in red, open Alix to activate it.

Alix exists in the cytosol in an inactive conformation, in which the PRR is folded across the V domain and forms an auto-inhibitory interaction with the Bro1 domain (Figure 3). This ‘closed’ conformation holds the Bro1 and V domains against each other [48], preventing Alix from engaging CHMP4B, while also blocking a conserved FYx_2_L motif [35,36] within the V domain from accessing other effectors [49]. Several mechanisms activate membrane-associated Alix, by opening the V domain and helping to re-orientate it with respect to the Bro1 domain (Figure 3). These mechanisms include: binding of the TSG101 subunit of ESCRT-I to the PRR, or binding of Src to a cognate-binding site within the Bro1 domain, to displace the auto-inhibitory interaction [49]; phosphorylation of the PRR [50]; allosteric relief of the auto-inhibition via the binding of CEP55 (centrosomal protein of 55 kDa) or Alg-2 to distal sites within the PRR [51,52]; dimerisation of the V domain [53].

In contrast with the widespread cellular roles of Alix, HD-PTP acts selectively at the endosome and is essential for the forward movement of EGFR from the early endosome towards the lysosome. Its depletion causes a striking accumulation of tubulo-vesicular endosomal compartments that contain a build-up of ubiquitin–protein conjugates and reduces sorting of EGFR to the endosomal lumen [43,54]. Consistent with a function in EGFR trafficking, HD-PTP binds the EGFR adaptor Grb2, via the PRR [55]. The importance of HD-PTP for MVB sorting is highlighted by its necessity for the down-regulation of platelet-derived growth factor receptor [56] and α5β1 integrin [57], as well as MHC (major histocompatibility complex) class I targeted by the K3 ubiquitin ligase of Karposi's sarcoma-associated herpes virus [58]. Consistent with a role in receptor down-regulation, HD-PTP is a tumour suppressor [39,59,60] and HD-PTP haploinsufficiency is linked to a poor clinical prognosis [61].

**Figure 4.**
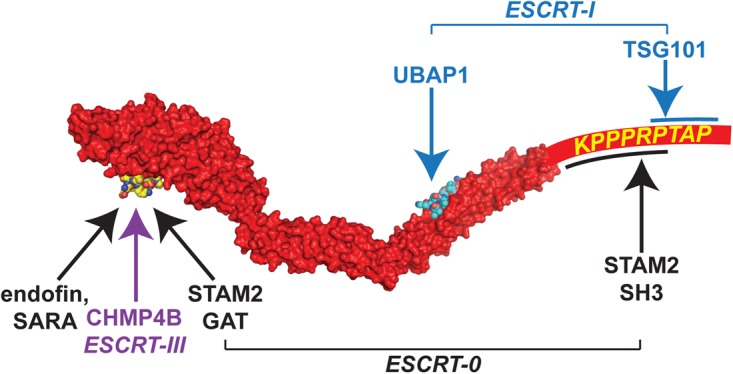
HD-PTP acts as an open ESCRT-binding scaffold. Model of the Bro1 and coiled-coil domains of HD-PTP based on crystal and solution structures, and extended to include the proximal region of the PRR. Bound UBAP1 and CHMP4B peptides are shown based on crystal structures.

HD-PTP works closely with ESCRT-0, and indeed is essential for releasing EGFR from ESCRT-0 and allowing it to engage ESCRT-III [54]. The ESCRT-0 subunit STAM2 (equivalent to yeast Hse1; see Table 1) binds directly to HD-PTP at two sites (Figure 4). Specifically, the STAM2 GAT (GGA and Tom1) domain binds at the CHMP4-binding pocket in the Bro1 domain [54,62], while the STAM2 SH3 domain binds to a peptide motif within the PRR [54]. An ESCRT-I complex, representing ∼10% of total ESCRT-I [63] and defined by the presence of UBAP1 (ubiquitin-associated protein 1) and VPS37A subunits, also co-operates closely with HD-PTP [63,64]. Consistent with their role in a specialised, endosome-specific ESCRT pathway [63], HD-PTP and UBAP1 are present in HeLa cells at relatively low copy number compared with several core ESCRT components including TSG101 and CHMP4B [65]. Like HD-PTP, UBAP1 is important for EGFR degradation and α5β1 integrin turnover [57,63], and is a candidate tumour suppressor [66,67]. Its C-terminal ubiquitin-binding region (SOUBA; solenoid of overlapping UBAs) [68] is essential for cargo trafficking [63]. UBAP1 binds to the conserved FYx_2_L motif within the coiled-coil domain of HD-PTP (Figure 4), but cannot bind Alix [69]. This binding selectivity is determined, in part, by the overall shape of each coiled-coil domain, which in HD-PTP adopts an open and extended conformation, and by local amino acid sequence differences within the respective FYx_2_L-binding pockets [69]. ESCRT-I also engages HD-PTP via TSG101 binding to the PRR [70], at a site that overlaps with that for the STAM2 SH3 domain [54]. The UBAP1-binding pocket is situated close by (Figure 4), so altogether the interactions of the ESCRT-0 SH3 domain and ESCRT-I with HD-PTP are most likely mutually exclusive.

HD-PTP adopts an open, extended platform for ESCRT binding that appears not to be subject to the auto-inhibition that in Alix is overcome by large-scale conformational changes [71]. Instead, access of the CHMP4B C-terminus to the HD-PTP Bro1 domain is governed by competitive interactions that would be well suited to HD-PTP's role as a regulator of endosomal trafficking and its action in moving EGFR forward from ESCRT-0 towards ESCRT-III [54] (Figure 4). One obvious distinction in the crystal structures of the CHMP4B-binding pockets of Alix and HD-PTP is that the latter is partially occluded in the absence of ligand, consistent with a lower association rate [72]. Hence, binding of CHMP4B to the cytosolic form of HD-PTP may be intrinsically unfavourable. Furthermore, the CHMP4-binding site in HD-PTP can specifically accommodate STAM2, by virtue of the STAM2 GAT domain occupying the CHMP4B-binding pocket and extending towards a neighbouring binding pocket [62] (termed the specific pocket, or S-site [72]) that is absent from Alix [62,72]. Two endosomal proteins that control TGFβ family receptor signalling, SARA (Smad anchor for receptor activation) and endofin, also bind selectively to HD-PTP in competition with CHMP4B (Figure 4), with their N-terminal helices each fully occupying both the CHMP4B and S-site [72]. In summary, STAM2, SARA and endofin could potentially modulate the MVB sorting of multiple receptors by preventing HD-PTP from binding to CHMP4B. Meanwhile, their regulated release could conceivably leave an opened binding pocket into which the CHMP4B C-terminal peptide is then accepted. This binding switch may explain how HD-PTP moves EGFR from ESCRT-0 towards ESCRT-III [54].

## A model for EGFR sorting

MVB sorting is conceptually challenging: ESCRTs attach to the cytoplasmic face of the endosome and sort cargo into vesicles that bud in the opposite direction, yet ESCRTs themselves somehow escape being packaged into the ILV. How might the HD-PTP pathway sort EGFR?

**Figure 5.**
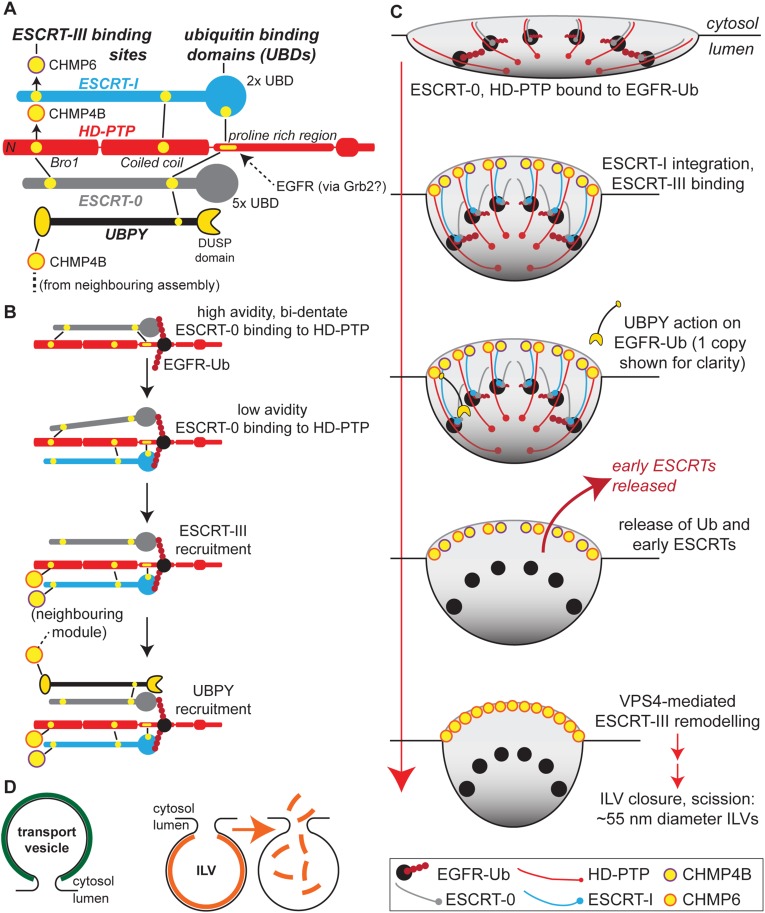
Identifying a potential mechanism for EGFR sorting to the MVB. (**A**) Alignment shows the polarity of the MVB sorting machine. Key binding sites are shown in yellow. HD-PTP binding to EGFR may occur via Grb2, but other modes of interaction are possible. (**B**) Competitive binding reactions may explain how HD-PTP supports the sequential assembly of ESCRTs upon ubiquitinated EGFR. (**C**) A speculative model, in which these competitive binding reactions are superimposed onto the pathway of ILV formation. HD-PTP and ESCRT-0 attach to a core of ubiquitinated EGFR in a radial configuration. ESCRT-I integration helps expose ESCRT-III-binding sites towards the rim and may generate membrane curvature. From ESCRT-III, UBPY reaches cargo to destabilise the assembly. (**D**) Solving a topographical problem. Transport vesicle formation requires an exoskeleton (coat) (left). An ILV might use an ESCRT endoskeleton that is triggered to disassemble prior to membrane scission (right).

HD-PTP forms an open console on which ESCRTs can shuffle via competitive interactions (Figures 4 and 5A). ESCRT-0 [13] and ESCRT-I [73] are elongated complexes. Aligning them with HD-PTP according to their bi-dentate binding reactions reveals a striking polarity to the machinery: UBDs converge near where HD-PTP engages EGFR, while ESCRT-III-binding sites are ∼20–30 nm away (Figure 5A). Such polarity extends to include the deubiquitinase UBPY (Ub-protease Y, equivalent to yeast Doa4; Table 1), which binds to both CHMP4B and to the STAM SH3 domain [74], and supports HD-PTP-dependent EGFR sorting [54,75]. Overall, this arrangement is difficult to reconcile with a linear, end-over-end pathway. However, it fits a model in which an HD-PTP scaffold recruits ESCRTs sequentially to ubiquitinated EGFR (Figure 5B). We speculate that a radial arrangement of HD-PTP molecules around an EGFR-rich membrane domain would create a configuration capable of imposing curvature (Figure 5C). This assembly would simultaneously generate sites for ESCRT-III monomers to attach towards the rim of a forming vesicle, in readiness for well-ordered polymerisation (Figure 5C). In this scenario, UBPY, bound to ESCRT-III, should have sufficient reach to deubiquitinate EGFR and thus destabilise the structure, triggering ILV involution as a prelude to membrane scission.

In summary, while budding of transport vesicles into the cytoplasm requires an *exoskeleton* (i.e. a ‘coat’), we envisage that ILV formation might be driven by an *endoskeleton* that is triggered to disassemble prior to the neck closing (Figure 5D). Though speculative, this simple model reconciles key features of MVB sorting, including the ordered action of ESCRTs, without invoking large-scale movement of cargo or ESCRTs themselves. Moreover, it provides a route for the potentially highly damaging ESCRT-III polymer [76] to assemble in an ordered fashion from pre-positioned ESCRT-III monomers, towards the rim of a developing ILV. Such a location is optimal for triggering ESCRT-III polymerisation [77]. Furthermore, the C-terminal region of HD-PTP has an extended PRR containing multiple SH3-binding sites that bind Grb2 and potentially other SH3 domain signalling proteins, as well as an important PTPase domain [39]. It could thus engage an EGFR signalling core, consistent with the marked recruitment of HD-PTP to endosomes upon EGFR activation [78]. This may allow ESCRT assembly, and thus MVB sorting, to be coupled to the signalling status of EGFR, a prerequisite for carefully orchestrated receptor down-regulation. Finally, such an arrangement of ESCRTs may also be relevant for other reverse-topology membrane scission events.

## Abbreviations

Alg-2: apoptosis-linked gene 2
Alix: ALG-2 interacting protein X
Bro1: Bck1-like resistance to osmotic shock 1
CHMP: charged membrane protein/chromatin modifying protein
EGFR: epidermal growth factor receptor
ESCRT: endosomal sorting complexes required for transport
GAT: GGA and Tom1
Hse1: Hrs-binding protein, STAM and EAST 1
ILV: intraluminal vesicle
MVB: multivesicular body
PRR: proline-rich region
SARA: Smad anchor for receptor activation
STAM: signal transducing adaptor molecule
TSG101: tumour susceptibility gene 101
UBD: ubiquitin-binding domain
UBPY: Ub-specific protease Y
Vps: vacuolar protein sorting
